# Allograft reconstruction of acetabular labrum has comparable outcomes to labral refixation

**DOI:** 10.1093/jhps/hnac053

**Published:** 2023-02-24

**Authors:** Mohammad S Abdelaal, Ryan M Sutton, Cenk Atillasoy, Javad Parvizi

**Affiliations:** Rothman Orthopaedic Institute at Thomas Jefferson University, 125 S 9th St. Ste 1000, Philadelphia, PA 19107, USA; Rothman Orthopaedic Institute at Thomas Jefferson University, 125 S 9th St. Ste 1000, Philadelphia, PA 19107, USA; Rothman Orthopaedic Institute at Thomas Jefferson University, 125 S 9th St. Ste 1000, Philadelphia, PA 19107, USA; Rothman Orthopaedic Institute at Thomas Jefferson University, 125 S 9th St. Ste 1000, Philadelphia, PA 19107, USA

## Abstract

The acetabular labrum plays an important role in hip stability, intra-articular fluid pressurization and force distribution. For irreparable labral pathology, labral reconstruction is an increasingly adopted technique shown to decrease hip pain and improve function. We evaluated survivorship and clinical outcomes of allograft labral reconstruction using the mini-open anterior surgical approach. Twelve patients who underwent labral reconstruction using a semitendinosus tendon allograft (reconstruction group) were matched 1:3 based on age, gender, body mass index, year of surgery, preoperative Tönnis grade, previous hip surgery, residual hip pathology and extent of acetabular chondral lesion to a control group of 36 patients who underwent direct labral repair with anchors (refixation group). At a minimum follow-up of 2 years, patient-reported outcomes, radiological findings and failure rates were compared. The average age was 31.3 years (±13.6) for reconstruction and 34.7 (±10.2) for refixation. Both groups had similar preoperative symptomatic periods (*P* = 0.3), prevalence of residual hip pathology (*P* = 1.0) and prevalence of prior hip surgeries (*P* = 1.0). both groups had a significant improvement of modified Harris Hip scores and 36-Item Short-Form Health Survey physical scores. There was no statistically significant difference in conversion rates to total hip arthroplasty (25% versus 8.3%, *P* = 0.2); however, time to conversion was significantly longer in the reconstruction group (3.58 years ±1.04) compared to the refixation group (1.20 years± 0.93; *P* = 0.04). In conclusion, at a minimum of 2 years of follow-up, mini-open labrum reconstruction for severe insufficiency of acetabular labrum demonstrated comparable improvements in functional outcomes and significantly longer survivorship compared to labral refixation.

## INTRODUCTION

The acetabular labrum serves an important role in hip function through deepening of the joint and resistance to lateral and vertical motion of the femoral head within the acetabulum [1]. The intact labrum helps to create a negative pressure within the hip joint, which maintains a fluid seal that is crucial for cartilage nutrition and protection [2, 3]. Tears in the labrum are associated with hip pain and alteration in the hip joint biomechanics [4, 5]. Labrum tears disrupt the suction seal, which can lead to cartilage delamination, early degeneration and potentially hip osteoarthritis (OA) [6–8].

Management options for labral tears have evolved over the years. Earlier experience involved segmental labral resection to achieve pain relief [9, 10]. Recently, with the improvement in our understanding of the labral role in hip stability and function, there has been growing interest in labral preservation, particularly in younger patients [7, 11]. Labral refixation with anchors has become the gold standard for managing labral tears during femoroacetabular impingement (FAI) surgeries [5]. However, severe labral defects or prior debridement can render the acetabular labrum unsalvageable. In such patients, acetabular labral reconstruction has shown promising early clinical outcomes as an alternative to complete resection [6, 9, 12]. It has the potential to re-establish the fluid seal in the hip joint [13, 14] and has led to improvements in symptoms, function and the satisfactory level of return to play among elite athletes [15].

There are still limited data on the optimal graft tissue, surgical approach or indication for labral reconstruction. Most labral reconstruction procedures are performed arthroscopically [16]; however, it is believed that there are still some indications for open techniques. Laboudie *et al.* recently published their results on labral reconstruction using open hip surgical dislocation and showed that labral reconstruction demonstrated comparable improvements of patient-reported outcomes (PROs) and similar survivorship to labral repair and debridement [17]. During standard open techniques, surgical hip dislocation is performed, and while it is considered safe and helps preserve femoral head vascularity, it is not without complications, including trochanteric non-union, bursitis, residual pain and decreased patient satisfaction [18, 19]. In our practice, we address FAI and labral pathologies, including reconstruction procedures, via a mini-open anterior hip approach. The minimally invasive approach has comparable efficiency to open hip dislocation but avoids its significant consequences [20, 21].

In the current study, we present our experience on the outcomes of allograft labral reconstruction using the mini-open approach. In order to better understand the contribution of labral reconstruction in this multi-step procedure, we compared the survival, clinical and radiological outcomes of labral reconstruction versus those of labral refixation in matched cohorts undergoing otherwise similar FAI surgeries.

## METHODS

After obtaining approval for data access from the institutional review board, we reviewed data from a prospectively maintained institutional database of all patients who underwent a mini-open FAI surgery at a single institution from 2003 to 2019. This database is being maintained by manually reviewing all patient charts and recording relative findings from office visit notes and operative reports, preoperative and postoperative radiographic measurements, outcome scores and any other relevant data. The general indications for FAI surgery were history and physical examination consistent with FAI, radiographic evidence of labral or chondrolabral tears, no or minimal arthritic changes and evidence of focal impingement (cam, pincer or both). Most labral reconstructions were performed in younger patients with FAI and no or minimal OA when the substance of labrum was inadequate for repair or when there was a disruption in the continuity of labrum that could not be restored. Inclusion criteria for the study and control groups included patients with symptomatic FAI without significant OA (Tönnis grade ≤2), who underwent mini-open surgery including femoroplasty and/or acetabuloplasty plus either labral reconstruction or labral refixation by the same surgeon and who underwent similar postoperative rehabilitation protocols. The minimum follow-up period for inclusion was 2 years.

Twelve patients who had irreparable labral defects that required labral reconstruction using semitendinosus tendon allograft (reconstruction group) met our inclusion criteria. We matched this cohort to a control group of 36 patients who underwent labral repair with anchors (refixation group). Matching was performed using propensity scoring on a scale of 1:3 based on age, gender, body mass index (BMI), year of surgery, preoperative Tönnis grade, previous hip surgery, residual hip pathology (slipped capital femoral epiphysis, severe dysplasia and Legg–Calves–Perthes disease) and extent of acetabular chondral lesion.

The primary outcome for this study was survivorship and time until failure, with the end-point defined as revision FAI surgery or conversion to total hip arthroplasty (THA). Secondary outcomes included PROs including the 36-Item Short-Form Health Survey (SF-36) score and the modified Harris Hip Score (mHHS). Also, radiographic parameters included preoperative and postoperative alpha angles [22] [on both anteroposterior (AP) and 45° Dunn’s lateral views], lateral center-edge angle (LCEA) [23] on the AP view, anterior center-edge angle (ACEA) [24] on the false-profile view, Tönnis angle [25] and superior and medial joint space. Findings related to acetabular retroversion (crossover sign [26], posterior wall sign [27] and ischial spine sign [28]) were also analyzed.

Furthermore, we compared the intraoperative findings between both cohorts. During the procedure, the senior author systemically graded articular cartilage damage of the acetabulum and femoral head via direct observation of the articular surface and assessment for cartilage softening, presence and depth of fissuring and fibrillation, chondral flaps and presence of exposed subchondral bone. Articular cartilage damage to the acetabulum and femoral head was graded as either partial thickness or complete thickness. Treatment intervention for cartilage defects included microfracture or debridement. The surgeon also recorded the pattern of labral abnormalities including tear, calcification, degeneration or detachment. Finally, modalities of labral management such as repair, partial resection, total resection and reconstruction were also documented.

### Surgical technique

**Fig. 1.**
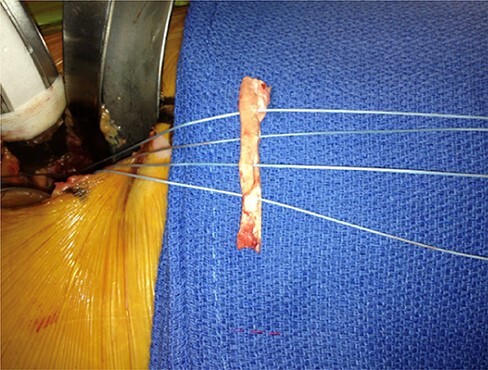
Preparation of the allograft for insertion with anchors.

Patients were positioned supine on a regular operating table. The knee was slightly flexed to relax the anterior structure of the hip (capsule, rectus femoris and psoas). A 3-cm longitudinal incision centered over the greater trochanter was performed distal to the anterior superior iliac spine. The superficial fascia of the thigh was opened along the tensor fasciae latae muscle which is partially detached from its aponeurosis and then retracted laterally. Superficially, the tensor muscle was retracted laterally and the sartorius muscle medially, and through this window, the rectus femoris muscle was retracted medially and gluteus medius muscle laterally to expose the hip capsule. Then, the surgeon performed an I-shaped capsulotomy to visualize and evaluate the cam deformity. At this time, the acetabular labrum was checked for the evidence of labral abnormality. When the labrum was found to be previously removed, the substance of the labrum was inadequate for repair, or when there was a disruption in the continuity of labrum that cannot be restored, labral reconstruction was performed. The acetabular rim was prepared by trimming of any acetabular overhang and resection of pathological labral tissue. Then, predrilling of all anchor sites was performed at 1-cm intervals using 3.2-mm drill holes. The allograft was then prepared and fixed to the acetabular rim using three or four anchors (Fig. 1). In the refixation group, labral repair was performed using anchor sutures secured into the acetabular rim (usually a minimum of three anchor sutures).

**Fig. 2.**
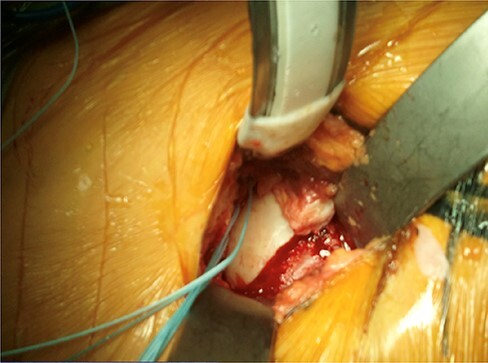
A Cobb retractor with a soft (rubber) cap was used to provide local distraction.

For a short time, traction was applied by an assistant to the lower limb in order to distract the joint for visualization. A Cobb retractor with soft (rubber) cap was used to hold local distraction (Fig. 2). Most of the articular surface could be seen and evaluated through this maneuver. A blunt tip hook was utilized to palpate the posteroinferior surface of the acetabulum which was not under direct visualization. Partial-thickness chondral lesions were excised, while full-thickness chondral lesions were managed via microfracture. In patients with acetabular overcoverage (pincer type), acetabuloplasty was performed. However, in patients with evidence of acetabular undercoverage, the surgeon performed very minimal refreshing of the acetabular rim to prepare the bed for later labral reattachment. Femoral head–neck junction osteoplasty and cam removal were performed using a 5-mm high-speed bur and small osteotome until an impingement-free hip range of motion was achieved. Without putting any traction on the lower extremity, we rotate the hip through its full range of motion, aiming for 30–50 degrees of internal rotation and 120–130 degrees of flexion. Additional osteoplasty was undertaken if the impingement-free range of motion was not achieved. In addition, the ability to have a direct visualization of the femoral neck provided excellent input in determining the adequacy of resection. Then, the joint capsule was closed meticulously in all patients with a running suture. We did not use fluoroscopy during the surgery. Postoperatively, protected weight-bearing was typically allowed in all patients for 2–4 weeks followed by full weight-bearing. Return to sports and unrestricted full activity normally took 4–6 months for most patients.

### Statistical analysis

Propensity scores generated using logistic regression were used to adjust for the aforementioned confounding variables in order to minimize selection bias and ensure covariate balance. Continuous data were presented as either the mean ± SD for parametric data or median with the associated range for non-parametric data. Categorical data were presented as a percentage with numerator and denominator in parentheses. An independent sample *t*-test was used for parametric data, and the Mann–Whitney *U* test for non-parametric data. The chi-square or Fisher exact test was used for categorical data. All statistical analyses were performed using RStudio (Version 3.6.1; Vienna, Austria).

## RESULTS

In our study, 12 patients who underwent FAI surgery with labral reconstruction with a mean follow-up of 6.4  (±1.49) years were matched to 36 patients with labral refixation with a mean follow-up of 6.68 years (±2.01) (*P* = 0.608). The reconstruction cohort had seven female patients and five male patients with a mean age of 31.3 (±13.6) years. The refixation cohort consisted of 36 patients, 16 females and 20 males, with a mean age of 34.7 (±10.2) years. Gender, BMI, mean preoperative symptomatic period, laterality, grade of acetabular chondral lesion and prevalence of residual hip pathology were comparable between groups. Four patients in the reconstruction cohort (33.3%) and eleven patients from the refixation cohort (30.6%) underwent prior FAI surgery on the ipsilateral hip (*P* = 1.0) (Table I).

**Table I.** Patient characteristics

	*Refixation group (n = 36)*	*Reconstruction group (n = 12)*	*P value*
Age, years	34.7 (10.2)	31.3 (13.6)	0.449
Gender			0.617
Female	16 (44.4%)	7 (58.3%)	–
Male	20 (55.6%)	5 (41.7%)	–
BMI	27.8 (5.45)	26.7 (4.08)	0.482
Operated hip	–	–	1.000
Left	14 (38.9%)	4 (33.3%)	–
Right	22 (61.1%)	8 (66.7%)	–
Preoperative symptomatic period	2.23 (2.49)	13.1 (33.1)	0.327
Follow-up	6.68 (2.01)	6.40 (1.49)	0.608
Surgeon experience	9.69 (2.14)	10.9 (1.80)	0.078
Residual hip diseases			
Severe dysplasia	3 (8.33%)	0 (0.00%)	0.563
Slipped capital femoral epiphysis	4 (11.1%)	2 (16.7%)	0.631
Legg–Calves–Perthes	1 (2.78%)	0 (0.00%)	1.000
Previous FAI surgery	11 (30.6%)	4 (33.3%)	1.000

Radiological measurements including preoperative and postoperative alpha angles (AP and Dunn’s view), Tönnis angle, superior and medial joint space width, LCEA, ACEA, crossover sign, posterior wall sign, ischial spine sign and hip congruency were similar between groups (Tables II, III). In the reconstruction group, five patients (41.7%) had no arthritic changes (Tönnis Grade 0), six patients (50.0%) had Tönnis Grade 1 findings and one patient (8.3%) had Tönnis Grade 2 osteoarthritic changes. In the refixation group, 13 patients (36.1%) showed no hip osteoarthritic changes (Tönnis Grade 0), 19 patients (52.8%) had Tönnis Grade 1 findings and four patients (11.1%) had Tönnis Grade 2 osteoarthritic changes.

**Table II.** Definitions of radiographic measurements

*Radiographic measurement [* 25 *]*	*Definition*
Alpha angles [22]	The angle formed by the axis of the femoral neck and a line drawn from the center of the femoral head to the point where prominence begins on the anterolateral head–neck junction
LCEA [23]	The angle formed by two lines: (I) a line through the center of the femoral head that is perpendicular to the transverse axis of the pelvis and (II) a line from the center of the femoral head that passes through the most superolateral point of the sclerotic weight-bearing zone of the acetabulum
ACEA [24]	The angle between a vertical line passing through the center of the femoral head and a line connecting the center of the femoral head with the most anterior point of the acetabular sourcil
Tönnis angle [25]	The angle between the inter-teardrop line (or two other reference points on each hemipelvis) and the weight-bearing dome (i.e., sourcil) of the acetabulum
Crossover sign [26]	It occurs when the anterior and posterior walls of the acetabulum meet caudal to the acetabular roof, so that the superior part of the anterior acetabular wall is lateral to (intersect) the superior part of the posterior wall
Posterior wall sign [27]	It occurs when the head center is located lateral to the posterior wall of the acetabulum
Ischial spine sign [28]	Exaggerated protrusion of the triangular-shaped ischial spine medially from the pelvic brim to the pelvic inlet

**Table III.** Radiological findings

	*Refixation group (n = 36)*	*Reconstruction group (n = 12)*	*P value*
Preoperative alpha (AP)	65.6 (18.1)	70.2 (21.0)	0.316
Preoperative alpha (Dunn’s, frog leg and cross-table)	76.0 (21.7)	59.7 (22.3)	0.546
Postoperative alpha (AP)	66.5 (26.9)	63.3 (20.3)	0.101
Postoperative alpha (Dunn’s, frog leg and cross-table)	8.12 (5.60)	6.43 (5.42)	0.976
Tönnis angle	6.80 (4.43)	7.55 (3.41)	0.394
Medial joint space	4.22 (1.20)	3.95 (1.23)	0.346
Superior joint space (mm)	28.4 (8.67)	30.6 (9.50)	0.317
LCEA	26.0 (6.77)	29.3 (5.20)	0.520
ACEA	26.0 (6.77)	29.3 (5.20)	0.259
Tönnis grade of OA	–	–	1.000
0	13 (36.1%)	5 (41.7%)	–
1	19 (52.8%)	6 (50.0%)	–
2	4 (11.1%)	1 (8.33%)	–
Crossover sign	12 (33.3%)	2 (16.7%)	0.465
Posterior wall sign	12 (33.3%)	4 (33.3%)	1.000
Ischial spine sign	9 (25.0%)	2 (16.7%)	0.705
Congruency	–	–	0.233
Congruent	18 (50.0%)	3 (25.0%)	–
Mildly incongruent	15 (41.7%)	8 (66.7%)	–
Incongruent	3 (8.33%)	1 (8.33%)	

Intraoperatively, the reconstruction group demonstrated full acetabular chondral lesions in 66.7% of cases and partial chondral lesions in 25% of cases, which were comparable to chondral lesions in the refixation group (75% and 16.7%, respectively, *P* = 0.854). In terms of management of these chondral lesions, 33.3% of patients in the reconstruction group underwent microfracture and 58.3% underwent mechanical debridement, which was similar to the rates of management of chondral lesions in the refixation group (44.4% and 47.2%, respectively, *P* = 0.880). Also, similar percentage of cases required acetabular trimming in both groups (*P* = 0.703). In terms of the types of labral abnormalities, four cases in the reconstruction group (33.3%) had labral tears compared to 36 cases in the refixation group (100%) (*P* < 0.001). Labral calcification was detected in five cases in the reconstruction group (41.7%) compared to no cases in the refixation group (*P* < 0.001). On several occasions, multiple simultaneous abnormalities were seen in the labrum (Table IV). Comparable improvement in the range of motion of the hip was seen between groups (Table V).

**Table IV.** Intraoperative findings

	*Refixation group (n = 36)*	*Reconstruction group (n = 12)*	*P value*
Acetabular chondral lesion			0.854
Full	27 (75.0%)	8 (66.7%)	–
Partial	6 (16.7%)	3 (25.0%)	–
Treatment chondral lesion	–	–	0.880
No treatment	3 (8.33%)	1 (8.33%)	–
Microfracture	16 (44.4%)	4 (33.3%)	–
Mechanical	17 (47.2%)	7 (58.3%)	–
Acetabular trimming	10 (27.8%)	2 (16.7%)	0.703
Labral abnormalities			
Labral tear	36 (100%)	4 (33.3%)	<0.001^*^
Labral calcification	0 (0.00%)	5 (41.7%)	<0.001^*^
Labral degeneration	4 (11.1%)	4 (33.3%)	0.094
Labral detachment	9 (25.0%)	1 (8.33%)	0.414

* Differnce is statistically significant.

**Table V.** Range of motion

	*Refixation group (n = 36)*	*Reconstruction group (n = 12)*	*P value*
Preoperative flexion	92.2 (4.41)	100 (14.1)	0.578
Postoperative flexion	110 (6.24)	116 (5.48)	0.087
Preoperative internal rotation	11.2 (7.91)	15.0 (7.07)	0.590
Postoperative internal rotation	35.6 (5.07)	33.3 (5.16)	0.363

In terms of failure rates, no cases required FAI surgery revision procedures after the reconstruction or refixation surgeries. THA conversion rates were higher in the reconstruction group (25% versus 8.3%); however, the difference was not statistically significant (*P* = 0.156) (Table VI). Interestingly, time to THA conversion was statistically longer in the reconstruction group compared to the refixation group [3.58 years (±1.04) versus 1.2 years (±0.93), *P*= 0.042].

**Table VI.** Outcomes

	*Refixation group (n = 36)*	*Reconstruction group (n = 12)*	*Odds ratio*	*P value*
Preoperative mHHS	53.6 (9.39)	56.7 (19.4)	1.03 [0.87–1.21]	0.864
Postoperative mHHS	69.8 (22.3)	85.8 (1.63)	1.08 [0.91–1.28]	0.184
Preoperative SF-36 physical	37.9 (6.46)	41.2 (7.91)	1.08 [0.90–1.30]	0.490
Postoperative SF-36 physical	38.6 (10.5)	47.8 (6.03)	1.12 [0.96–1.30]	0.067
Failure				
Conversion to THA	–	–	–	0.156
No	33 (91.7%)	9 (75.0%)	Ref.	–
Yes	3 (8.33%)	3 (25.0%)	3.54 [0.53–23.9]	–
Time to failure (years)	1.20 (0.93)	3.58 (1.04)	–	0.042^*^

* Differnce is statistically significant.

With the numbers of scores available, improvements in mHHS and SF-36 scores were statistically significant in both groups at the 2-year postoperative follow-up (*P* < 0.001). There was no postoperative difference in the mean ± SD mHHS between the reconstruction group and the refixation group (85.8 ± 1.63 points versus 69.8 ± 22.3 points, *P* = 0.184). The change in the mHHS (Δ) was larger in the reconstruction group, with a +29.1-point increase, compared to the refixation group, which had a +16-point increase, but not statistically significant. There was also no significant postoperative difference between groups regarding the physical component of SF-36 scores (47.8 ± 6.03 points versus 38.6 ± 10.5 points, *P* = 0.067) (Table VI).

## DISCUSSION

This study is the first to the authors’ knowledge to evaluate the results of employing allograft transplant for acetabular labral reconstruction via the mini-open anterior approach to the hip. Labral reconstruction aims to regenerate the labral seal in order to restore labral function, improve joint lubrication and improve hip mechanics in patients with severe labral insufficiency [29]. Our results demonstrate that labral reconstruction can provide comparable clinical and radiological outcomes to labral refixation. Furthermore, labral reconstruction provided longer survivorship than labral refixation.

To better understand the effectiveness of this intervention for the management of irreparable labrum damage, we employed a comparative group of salvageable labral lesions treated with refixation. Although matching our study group to a similar control group of patients who had total labral resection without replacement would have provided a presumably ideal comparison, our database demonstrated that patients with resection were significantly older and had more osteoarthritic changes which would create a very different pool of patients and would not allow for valid comparison. It is understandable that patients who underwent reconstruction had non-functional or severely damaged labra as compared with patients who were treated with refixation. Our hypothesis was that comparing patients with labral reconstruction to a gold standard of management of labral tears (i.e. refixation) would give us insight into the efficacy of this intervention in the treatment of severely damaged labra, especially when groups are controlled for variables such as previous hip pathology, extent of acetabular chondral lesion and prior surgeries.

Our results support the findings of previous studies which reported on outcomes of labral reconstruction compared to labral refixation. Chandrasekaran *et al.* [30] compared 34 cases of labral damage treated by reconstruction using ipsilateral gracilis autograft or semitendinosus allograft to a matched group of 68 patients treated with refixation of their labral tears. They reported no significant differences in pre- and postoperative PROs, visual analog scale (VAS) scores and patient satisfaction between both treatment options. Furthermore, there was no significant difference in the conversion to THA or incidence of reoperations. Matsuda and Burchette [14] compared eight cases of acetabular labral reconstruction using gracilis autograft to 46 cases of labral refixation, with greater improvement of PROs in the reconstruction group, although the baseline preoperative scores were significantly lower compared to the refixation group.

Proper selection of patients for labral reconstruction is essential. Poor outcomes and ultimate conversion to a THA depend on several important factors, regardless of labral treatment. Older patients, patients with more significant degenerative joint disease and patients with a joint space <2 mm are more likely to progress to THA [31]. To optimize outcomes after labral reconstruction, these factors should be taken into account when selecting patients for surgery. Philippon *et al*. [10] published a study on labral reconstruction using autograft in 47 patients, and almost half of them had a history of previous surgeries to the involved hip. Patients younger than 30 years had better outcomes and satisfaction, and patients with a joint space width of <2 mm had lower satisfaction scores.

The rate of conversion to THA in our reconstruction group (25%) was consistent with previous studies. Geyer *et al.* reported on 76 cases of labral reconstruction using an iliotibial band autograft [12] and with a conversion rate of 23.7% at 2.33 years. In our study, survivorship time was longer (3.58 years). White *et al.* found that patients undergoing labral repair in the setting of revision surgery were 2.6 times more likely to fail, as defined by subsequent intra-articular hip surgery compared to labral reconstruction with no statistically significant difference in outcome scores or VAS scores between reconstruction and repair patients [32].

There is a wide variation of techniques and graft options to replace unsalvageable labral damage. Our results showed that semitendinosus allograft is a relatively safe procedure with lower morbidity for the patient. Suggested advantages of allograft reconstruction include avoidance of donor site morbidity, risk of nerve injury, wound-healing complication and infection that may be associated with autograft harvesting [6, 14]. Also, allograft utilization is associated with decreased surgical time and enhanced control over graft size and composition [31, 33]. However, the high cost of utilizing an allograft continues to be a limitation factor compared to autologous graft in soft tissue reconstruction [34].

Our findings demonstrated that patients in the reconstruction group had higher improvements in the mHHS compared with the matched refixation group despite similar baseline scores. However, this difference did not reach statistical significance, possibly due to the small sample size of the reconstruction group, which may be underpowered to adequately reveal the magnitude of effects. Another explanation for this finding is the symptom relief associated with the resection of the damaged labrum with accompanying nociceptors and replacement with graft with no nerve supply [35]. Nevertheless, these results showed that the reconstruction cohort had, at a minimum, comparable outcomes to the refixation cohort, which supports the contributory effect of allograft labral reconstruction in managing irreparable labrum damage.

The main strengths of this study are the inclusion of a matched control group with similar baseline demographic characteristics, Tönnis grade of cartilage degeneration, previous hip surgery, residual hip pathology and extent of intraoperative acetabular chondral lesion. Controlling for these variables strengthens comparative analysis and helps to evaluate the contribution of labral reconstruction in patients with FAI undergoing similar procedures by the same surgeon following the same postoperative protocols and similar follow-ups during the same time period. Limitations of this study include the small sample size; however, we believe that the small size reflects the relative infrequency of cases with unsalvageable labral damage. Another limitation for this study is its retrospective nature, although data were prospectively collected, which reduces recall bias and limits selection bias. Other limitations were related to the length of the follow-up. Although the average follow-up time in this study was 6.4 years, it is unknown if this duration is adequate to fully capture long-term effects of labral reconstruction on the hip joint in this specific population. Furthermore, there was no objective assessment of labrum healing or intra-articular status utilizing magnetic resonance imaging or other imaging modalities. Finally, this is a single-surgeon case series, which limits to generalizability of the findings in this study.

## CONCLUSION

Mini-open labral reconstruction with allograft is associated with improved survivorship and comparable outcomes to a matched control group treated with labral repair. No differences were seen with respect to need for secondary surgery or conversion to THA. Labral reconstruction appears to serve as a viable option for the treatment of unsalvageable labral tears; however, further evaluation in larger studies with long-term follow-up is needed.

## Data Availability

The data utilized for this study are not publicly available.
